# Leadership and Management in Integrated Services

**Published:** 2013-09-25

**Authors:** Lorna Roe

**Affiliations:** Health Research Boards SPHERE Programme (Roe's current project can be found in https://medicine.tcd.ie/health_policy_management/assets/pdf/SPHeRE-Brochure-2013.pdf), Centre for Health Policy and Management in Trinity College Dublin, Dublin2, Ireland

In this book Judy McKimm and her colleagues put flesh on the theoretical skeleton of an integrated care approach by looking at the concepts of leadership and management, and how they can significantly add to one's appreciation of integrated care.

The book looks at concepts, models and theories of management, leadership and team-working and their applicability within an integrated care approach to health and social care delivery. It considers the legal and policy landscape of integrated care and the limit of its reach in changing practice on the ground; leadership styles, their applicability, use and effect; personality traits, features and benefits of an emotionally intelligent professional in an integrated team; and professional roles, education and training curricula involved in shaping the professional behaviours necessary to working in an integrated environment.

A key strength of this book is its efficacy in seeing the whole picture of an integrated care approach. It leads the reader through a diverse range of simultaneously occurring factors which drive home the complex nature of the integrated care agenda.

Chapters begin with a thorough and accessible discussion on each topic and its role in integrated care. Each component is usefully complemented with illustrative case studies across a range of sectors from children, mental health and older people, showing theories and principles in practice. While the case study literature is notably Britain-centric, which makes it less amenable to an international audience, the examples provided are well described and deliver useful learning on initiatives and their effects in the UK context.

One of the key lessons you will take from this book is the reflexive, sensitive and smart skills that are required to successfully deliver an integrated service. Chapters 1 and 3 discuss leadership styles and their impact, particularly drawing the reader to two conclusions. Firstly, one size will not fit all situations; some issues will warrant a coaching leader where others might require a democratic leader and secondly, situations are in a constant state of development and require a perceptive and flexible leader to evolve alongside the workplace and its staff.

The articulation of ‘deliberate strategy’ (top-down) and ‘emergent strategy’ (bottom-up) is illuminating in showing how different paths can be taken to develop integrated care. Equally the book offers numerous examples of models of integrated care, each with its own function such as project partnerships, problem-oriented partnerships and ideological partnerships.

Chapters 5–7 delve into the psychology of human behaviour, unearthing known responses to change, which is highly relevant for staff facing working in an integrated manner where previously they worked in familiar and independent professional silos. The concept of ‘attractor patterns’ in response to innovation is particularly relevant. It states that individuals evaluate and assess potential change from their own perspective, which leads to individual and (subsequently) group resistance or acceptance of change. It is particularly applicable when one considers the political landscape that must be travelled in order to attain a consensus of individuals constructively reaching agreement on working together.

A slightly distracting feature of the book is the manner in which it repeats previously covered literature. However, it is worth sticking with and reading through as, while repetitive, it will drive home how all the factors are linked and mutually occurring.

So who should buy this book? This book will not be for everyone. While it fits an educational niche with its comprehensive overview of leadership and management within integrated care, it fails to thoroughly discuss the concept and goals of integrated care which will make it less insightful to readers new to the body of literature on integrated care.

Similarly, its diagnostic approach to dilemmas inherent within an integrated approach makes it of limited use to readers seeking a theoretical or evidence-based discussion on solutions to these problems.

Rather, the essence of this book lies in stimulating thought on the potential complexity in delivering integrated care and why it is a complex phenomenon. As such I would recommend it to students, practitioners and academics working in the fields of health and social care, health policy, health economics and health care management who are interested in deepening their understanding of the delivery of integrated care. I have emerged from reading it with a healthier respect and awareness of what it takes to deliver integrated care.

